# Cell-Based Therapies for Type 1 Diabetes: A Narrative Review of Stem Cell-Derived Islets, Encapsulation Strategies, and Recent Clinical Trials

**DOI:** 10.7759/cureus.111930

**Published:** 2026-07-01

**Authors:** Sruthi Guttikonda, Jai C Machur, Chinmai P Matha, Rhea R Mathew, Tushar Thotakura

**Affiliations:** 1 Medicine, Sri Manakula Vinayagar Medical College and Hospital, Kalitheerthalkuppam, IND; 2 Internal Medicine, Dr. D. Y. Patil Medical College, Hospital & Research Centre, Pune, IND; 3 Psychiatry, M.S. Ramaiah Medical College, Bengaluru, IND; 4 Medicine, St. Martinus University, Willemstad, CUW; 5 Medicine, Sri Ramachandra Institute of Higher Education and Research, Chennai, IND

**Keywords:** cell-based therapy, cell encapsulation, diabetes mellitus type 1, immunoisolation, induced pluripotent stem cells, islets of langerhans transplantation, stem cell-derived islets, type 1 diabetes

## Abstract

Type 1 diabetes (T1D) is an autoimmune disease characterized by the destruction of insulin-producing pancreatic β-cells, necessitating lifelong insulin therapy. While islet transplantation has demonstrated efficacy in restoring glycemic control, donor scarcity and the requirement for chronic immunosuppression limit its widespread application. Recent advances in stem cell biology, bioengineering, and immunomodulation have catalyzed a paradigm shift toward stem cell-derived β-cell replacement therapies. This narrative review presents recent clinical trials and technological innovations in cell-based therapies for T1D, with particular emphasis on stem cell-derived islet generation, encapsulation strategies for immunoprotection, and emerging clinical evidence. Landmark clinical trials and pioneering work with chemically induced pluripotent stem cells have demonstrated proof-of-concept for insulin independence and glucose-responsive C-peptide secretion. Encapsulation technologies, ranging from macroencapsulation devices to microencapsulation with bioactive materials, aim to eliminate the need for systemic immunosuppression while protecting grafts from immune rejection. Despite promising early results, challenges including fibrosis, vascularization, long-term graft survival, and scalable manufacturing remain. This review provides a comprehensive overview of the current state of cell-based therapies for T1D and outlines future directions for translating these innovations into routine clinical practice.

## Introduction and background

Introduction

Type 1 diabetes (T1D) is a chronic autoimmune disorder characterized by the selective destruction of insulin-producing pancreatic β-cells, resulting in absolute insulin deficiency and lifelong dependence on exogenous insulin administration [1]. Despite significant advances in insulin delivery technologies and continuous glucose monitoring systems, many patients continue to experience glycemic variability, severe hypoglycemia, and long-term complications, including cardiovascular disease, nephropathy, and retinopathy [2]. The restoration of endogenous insulin production through β-cell replacement represents a potentially curative approach that could fundamentally transform T1D management.

Islet transplantation, pioneered by the Edmonton Protocol in 2000, demonstrated that transplanted donor islets could restore insulin independence and protect against severe hypoglycemia in select patients [3]. However, this approach is severely limited by the scarcity of deceased donor pancreata and the requirement for lifelong systemic immunosuppression, which carries significant risks, including increased susceptibility to infections, malignancies, and nephrotoxicity [4]. These limitations have catalyzed intensive research into alternative sources of insulin-producing cells, with pluripotent stem cell-derived β-cells emerging as the most promising solution.

Recent years have witnessed remarkable progress in generating functional β-cells from human embryonic stem cells (ESCs) and induced pluripotent stem cells (iPSCs), with several differentiation protocols now capable of producing glucose-responsive insulin-secreting cells at scale [5,6]. Concurrently, advances in bioengineering have yielded sophisticated encapsulation technologies designed to immunoprotect transplanted cells without systemic immunosuppression [7,8]. Multiple clinical trials have now demonstrated proof-of-concept for stem cell-derived β-cell therapies, with landmark studies reporting insulin independence and restoration of glucose-responsive C-peptide secretion in patients with T1D [9,10].

This narrative review provides a comprehensive overview of recent advances in cell-based therapies for T1D, focusing on three interconnected domains: (1) methods for generating functional β-cells from pluripotent stem cells, (2) encapsulation strategies and immunoprotection technologies, and (3) clinical trial outcomes demonstrating safety and efficacy. By integrating findings from recent clinical trials with technological innovations in stem cell biology and bioengineering, this review aims to provide a holistic perspective on the current state and future trajectory of cell replacement therapies for T1D.

Methods: Literature search strategy

This narrative review was conducted to synthesize recent advances in cell-based therapies for T1D, focusing on stem cell-derived islets, encapsulation strategies, and clinical trial outcomes. We employed a systematic and transparent literature search strategy to identify relevant publications. The literature search was conducted on February 05, 2025, across major electronic databases: PubMed and Scopus. The search strategy was developed iteratively with input from all co-authors to ensure coverage of three key domains: stem cell sources (embryonic stem cells, induced pluripotent stem cells, and chemically induced pluripotent stem cells), encapsulation technologies (macroencapsulation, microencapsulation, and biomaterial approaches), and clinical outcomes (safety, efficacy, C-peptide secretion, and insulin independence).

The primary search string combined MeSH terms and keywords related to T1D with terms for stem cell-derived islets, encapsulation and immunoprotection strategies, and clinical trials, using Boolean operators to refine the search appropriately for each database. The search terms included "type 1 diabetes", "T1D", and "diabetes mellitus type 1" combined with "stem cell-derived islets", "pluripotent stem cell", "ESC", "iPSC", "induced pluripotent stem cell", "embryonic stem cell", "CiPSC", "chemically induced pluripotent stem cell", "beta cell replacement", and "pancreatic progenitor". These were further combined with terms related to immunoprotection, including "encapsulation", "macroencapsulation", "microencapsulation", "immunoisolation", "immunoprotection", "hypoimmune", and "immune evasion". Finally, the search incorporated clinical terminology, including "clinical trial", "clinical study", "human", "patient", "transplantation", and "graft survival".

We established clear inclusion and exclusion criteria to guide study selection and ensure the relevance and quality of included publications. Studies were included if they involved human subjects with T1D or presented high-quality preclinical data in animal models evaluating translational strategies, if they reported on cell-based therapies, including stem cell-derived β-cells, encapsulated islets, or genetically engineered hypoimmune cells, and if they provided data on safety outcomes, glycemic control measures, C-peptide secretion, insulin requirements, or immunoprotection. We limited our search to English-language publications and prioritized studies published between 2015 and February 2026 to capture recent advances. Studies were excluded if they focused exclusively on insulin delivery technologies without cell-based interventions, reported only on mesenchymal stem cell therapies without β-cell replacement components, were case reports with fewer than three subjects, were conference abstracts without full-text publications, or were editorial or opinion pieces without original data. We also performed manual reference list screening of included articles and relevant review papers to identify additional studies not captured by the primary search. Data from included studies were extracted using a standardized form that captured study characteristics, intervention details, participant numbers, endpoints, and outcomes, with key findings synthesized narratively and summarized in a comparative table to facilitate cross-study analysis.

We acknowledge several inherent limitations of our search methodology. The rapidly evolving nature of the field means that new clinical data may emerge after our search date, and publication bias remains a concern, as positive findings are more likely to be published than negative or neutral results. The narrative synthesis approach, while appropriate for providing a broad overview of this complex and multifaceted field, does not permit quantitative meta-analysis, and the restriction to English-language publications may have excluded relevant non-English studies.

## Review

Background and theoretical foundations

The Rationale for Cell Replacement Therapy in T1D

The fundamental pathophysiology of T1D, autoimmune destruction of pancreatic β-cells, provides a clear rationale for cell replacement as a curative strategy. Unlike symptomatic management with exogenous insulin, which cannot fully replicate the dynamic, glucose-responsive insulin secretion of native β-cells, cell replacement therapy aims to restore physiological glycemic control [11]. A phase 3 clinical trial involving 15 non-pregnant adults aged 18-65 years who underwent deceased-donor islet transplantation documented near-normoglycemia, glycemic stability, and protection from severe hypoglycemia with an acceptable safety profile, validating the therapeutic potential of β-cell replacement [3].

However, the limited availability of donor islets, with only approximately 3,000 deceased donors annually in the United States, means that conventional islet transplantation can serve only a small fraction of the estimated 1.6 million Americans with T1D [12]. Furthermore, the requirement for chronic immunosuppression to prevent allograft rejection introduces significant morbidity, limiting the applicability of this approach to patients with severe hypoglycemic unawareness or brittle diabetes [13]. These constraints have driven the search for renewable sources of β-cells and strategies to eliminate or minimize immunosuppression requirements.

Evolution From Donor Islet Transplantation to Stem Cell-Derived Approaches

The field of cell replacement therapy for T1D has evolved through several distinct phases. The Edmonton Protocol, introduced in 2000, established the feasibility of islet transplantation using a glucocorticoid-free immunosuppressive regimen, achieving insulin independence in the majority of recipients (immunodeficient Fox Chase severe combined immunodeficient (SCID)/Beige mice) [14]. However, long-term follow-up revealed progressive graft dysfunction, with most patients requiring resumption of insulin therapy within five years, highlighting the need for improved approaches [15].

The discovery of methods to differentiate human ESCs into pancreatic lineages in the early 2000s, followed by the development of iPSC technology in 2006, opened new avenues for generating unlimited quantities of β-cells [16]. Early differentiation protocols produced immature pancreatic progenitors that required in vivo maturation, but iterative refinements have progressively improved the efficiency and maturity of stem cell-derived β-cells [5]. Recent protocols can generate cells that closely resemble native human β-cells in terms of gene expression profiles, ultrastructure, and glucose-stimulated insulin secretion [6].

Parallel advances in bioengineering have focused on developing immunoprotection strategies that could eliminate the need for systemic immunosuppression. Encapsulation technologies, both macro- and microencapsulation, aim to create a semipermeable barrier that allows nutrient and oxygen diffusion while preventing immune cell infiltration [17]. More recently, genetic engineering approaches have been employed to create "hypoimmune" or "immune-evasive" stem cells through targeted deletion or modification of human leukocyte antigen (HLA) genes [18]. These complementary technological advances have converged to enable the recent wave of clinical trials demonstrating proof-of-concept for stem cell-derived β-cell therapies.

Stem cell-derived islet generation: Methods and advances

Differentiation Protocols: From Pluripotent Stem Cells to Functional β-Cells

The generation of functional β-cells from pluripotent stem cells requires recapitulating the complex developmental program of pancreatic organogenesis. This process involves sequential differentiation through definitive endoderm, primitive gut tube, posterior foregut, pancreatic progenitors, and finally endocrine precursors that mature into hormone-producing cells [5]. Contemporary differentiation protocols typically employ stage-specific growth factors, small molecules, and culture conditions to guide cells through these developmental transitions over three to five weeks in vitro.

A critical challenge in stem cell-derived β-cell generation has been achieving functional maturity comparable to native adult β-cells. Early protocols produced cells that secreted insulin but lacked robust glucose responsiveness and exhibited immature gene expression profiles [19]. Recent advances have addressed these limitations through several strategies, including extended culture periods, three-dimensional aggregation into islet-like clusters, and incorporation of maturation-promoting factors [6]. Some protocols now generate cells that exhibit biphasic glucose-stimulated insulin secretion, appropriate expression of key β-cell transcription factors (PDX1, NKX6.1, MAFA), and ultrastructural features characteristic of mature β-cells [5].

An alternative approach involves transplanting pancreatic progenitor cells that undergo in vivo maturation within the recipient. This strategy, employed in several clinical trials, capitalizes on the host microenvironment to drive terminal differentiation [9]. While this approach has demonstrated clinical efficacy, it requires extended periods (months) for functional β-cell mass to develop and carries theoretical risks of incomplete differentiation or off-target cell fates [20].

ESCs vs. iPSCs

Both ESCs and iPSCs have been successfully employed to generate insulin-producing cells, with each cell source offering distinct advantages and challenges. ESCs, derived from the inner cell mass of blastocyst-stage embryos, represent the gold standard for pluripotency and have been extensively characterized in differentiation protocols [21]. The majority of clinical trials to date have utilized ESC-derived cells, reflecting their established track record and regulatory precedent [9,22].

iPSCs, generated through reprogramming of somatic cells, offer the theoretical advantage of autologous cell therapy, potentially eliminating the need for immunosuppression [23]. However, the generation of clinical-grade iPSC lines is time-consuming and expensive, and concerns about genomic instability and epigenetic memory have tempered enthusiasm for patient-specific approaches [24]. Recent studies have demonstrated that iPSC-derived β-cells can achieve functional outcomes comparable to ESC-derived cells, and several clinical trials are now evaluating iPSC-based therapies [10].

Comparative studies have generally found that ESC- and iPSC-derived β-cells exhibit similar differentiation efficiency and functional characteristics when subjected to optimized protocols, though line-to-line variability remains a consideration for both cell types [25]. The choice between ESC and iPSC sources may ultimately depend on factors such as regulatory considerations, manufacturing scalability, and the specific immunoprotection strategy employed.

CiPSCs: A Novel Approach

A groundbreaking development in stem cell-derived β-cell therapy has been the use of chemically induced pluripotent stem cells (CiPSCs), which are generated through chemical reprogramming rather than genetic manipulation [10]. This approach offers potential advantages in terms of safety, as it avoids the integration of reprogramming factors that could pose oncogenic risks. In 2024, a landmark clinical trial reported the first functional cure of T1D using autologous CiPSC-derived islets transplanted beneath the abdominal anterior rectus sheath [10].

The patient in this phase I trial achieved sustained insulin independence 75 days post-transplantation, with time-in-target glycemic range increasing from 43.18% to 96.21% by month four and exceeding 98% at one year [10]. Glycated hemoglobin decreased to non-diabetic levels (approximately 5%), and all study endpoints were met at one year without transplant-related abnormalities [10]. This remarkable outcome demonstrates the potential of autologous CiPSC-derived therapies to achieve durable glycemic control, though the requirement for immunosuppression in this trial limits insights into autoimmune responses against the transplanted cells [7].

The CiPSC approach represents a significant advance in personalized regenerative medicine for T1D, though questions remain regarding scalability, manufacturing timelines, and cost-effectiveness compared to allogeneic approaches. Further clinical trials will be essential to determine whether the benefits of autologous therapy justify the additional complexity and expense of patient-specific cell generation.

Encapsulation strategies and immunoprotection technologies

Encapsulation strategies and immunoprotection technologies are pivotal in advancing cell-based therapies for T1D. These approaches aim to protect transplanted insulin-producing cells from the host’s immune system, thereby reducing or eliminating the need for lifelong immunosuppression. Recent advancements in biomaterials and immunoengineering have significantly enhanced the potential of these therapies.

Macroencapsulation Devices

Macroencapsulation devices represent one of the most extensively studied approaches to immunoprotection of transplanted β-cells. These devices consist of semipermeable membranes that enclose large numbers of cells (typically millions to hundreds of millions) in a single retrievable unit [26]. The membrane pore size is designed to allow bidirectional diffusion of nutrients, oxygen, glucose, and insulin while excluding immune cells and antibodies [27].

Several macroencapsulation devices have advanced to clinical testing. The ViaCyte PEC-Encap device, a planar macroencapsulation system, was evaluated in early clinical trials for subcutaneous implantation of ESC-derived pancreatic progenitor cells [9]. While these trials demonstrated safety and proof-of-concept for in vivo maturation of transplanted cells, efficacy was limited by inadequate vascularization and fibrotic overgrowth of the device, which impaired nutrient and oxygen exchange [28]. These findings highlighted the critical importance of device design and implantation site selection for macroencapsulation success.

To address vascularization limitations, modified macroencapsulation devices incorporating vascular portals or prevascularization strategies have been developed [5]. These devices include features designed to promote host blood vessel ingrowth while maintaining immunoprotection [29]. Preclinical studies have demonstrated that macroencapsulation devices with portals support more rapid C-peptide release and improved cell survival compared to fully enclosed devices [5]. However, the introduction of vascular portals may compromise immunoprotection, necessitating careful optimization of pore size and membrane architecture.

Microencapsulation Approaches

Microencapsulation involves encasing small clusters of cells (typically 1,000 to 5,000 cells) within individual spherical capsules, typically 300 to 800 μm in diameter [30]. This approach offers several theoretical advantages over macroencapsulation, including improved nutrient and oxygen diffusion due to shorter diffusion distances, reduced foreign body response due to smaller individual capsule size, and the ability to distribute capsules throughout a larger tissue volume [31].

Alginate, a naturally derived polysaccharide, has been the most extensively studied biomaterial for microencapsulation due to its biocompatibility, mild gelation conditions, and tunable permeability [32]. Recent advances have focused on modifying alginate capsules with bioactive molecules to enhance function and reduce fibrosis. A notable example is the incorporation of CXCL12, a chemokine that promotes angiogenesis and may modulate local immune responses [6]. In a preclinical study in non-human primates, human stem cell-derived islets microencapsulated with CXCL12-modified alginate demonstrated functional survival for up to six months without systemic immunosuppression [6].

The study reported that the treatment was safe, with the healthy non-human primate maintaining stable C-peptide and blood glucose levels, while the diabetic animal showed detectable C-peptide for 13 weeks and reduced exogenous insulin requirements [6]. Retrieved capsules were predominantly free-floating without pericapsular fibrotic overgrowth, though cell viability decreased over time [6]. These findings suggest that bioactive modifications of encapsulation materials may enhance long-term graft function, though further optimization is needed to maintain cell viability.

Another promising approach involves coating capsules with biocompatible polycations to reduce immunogenicity and fibrosis. A preclinical study demonstrated that resized stem cell-derived β-cell clusters (approximately 150 μm diameter) combined with an A10 polycation coating induced long-term euglycemia (six months) in diabetic immune-competent mice [13]. After retrieval, these capsules showed minimal fibrosis and enhanced markers of β-cell maturation [13]. This strategy of optimizing both cell cluster size and capsule coating represents a promising direction for improving microencapsulation outcomes.

Biomaterials and Bioactive Modifications

Beyond alginate, a diverse array of biomaterials has been explored for cell encapsulation and immunomodulation. Polyethylene glycol (PEG)-based hydrogels offer tunable mechanical properties and can be functionalized with bioactive peptides or proteins [33]. Conformal coating with PEG has been evaluated as a strategy to create a thin immunoprotective layer around islets while minimizing diffusion barriers [34].

A particularly innovative approach involves microporous annealed particle hydrogels functionalized with immunomodulatory molecules [11]. In a preclinical study, microporous annealed particle hydrogels incorporating anti-CD3 monoclonal antibodies created a localized immunomodulatory microenvironment that protected stem cell-derived β-cells from autoreactive T cells [11]. These hydrogels supported rapid vascularization, minimal foreign body response, and successful engraftment of syngeneic islets in mice, with the anti-CD3 modification halting T cell migration [11]. This approach of combining physical encapsulation with active immunomodulation represents a promising strategy for addressing both alloimmune and autoimmune rejection.

Other bioactive modifications under investigation include incorporation of immunosuppressive drugs (e.g., rapamycin), anti-inflammatory cytokines, or molecules that promote vascularization, such as vascular endothelial growth factor [35]. The challenge lies in achieving sustained release kinetics that provide long-term protection without systemic effects or local toxicity. Advances in controlled-release technologies and stimuli-responsive materials may enable more sophisticated approaches to localized immunomodulation.

Hypoimmune Cell Engineering

An alternative or complementary approach to physical encapsulation involves genetically engineering stem cells to evade immune recognition. This strategy typically involves deletion or modification of HLA class I and class II genes, which are the primary targets of alloimmune responses [36]. Some approaches also incorporate the expression of immunomodulatory molecules such as PD-L1 or CD47 to actively suppress immune responses [37].

Recent reviews have highlighted the potential of hypoimmune stem cell engineering to overcome immune barriers and eliminate the need for immunosuppression [9,15]. However, concerns remain regarding the safety of HLA-deleted cells, particularly their potential susceptibility to natural killer cell-mediated lysis and the theoretical risk of malignant transformation in cells lacking immune surveillance [38]. Strategies to address natural killer cell recognition, such as expression of HLA-E or other natural killer cell inhibitory ligands, are under active investigation [39].

The combination of hypoimmune cell engineering with encapsulation technologies represents an emerging frontier that could provide synergistic immunoprotection. By reducing the immunogenicity of the transplanted cells while providing a physical barrier, this dual approach may achieve more robust and durable graft survival than either strategy alone [40,41]. Several clinical trials are now evaluating gene-edited immune-evasive cells, which will provide critical data on the safety and efficacy of this approach in humans [2].

Recent clinical trials: Safety, efficacy, and outcomes

Recent clinical trials have shown promising advancements in the use of stem cell-derived islets for treating T1D (Table 1). The potential of these therapies lies in their ability to replace the insulin-producing β cells lost in T1D, offering a regenerative approach to managing the disease. The trials have demonstrated encouraging results, such as the reduction or elimination of exogenous insulin administration and the detection of endogenous C-peptide in recipients, indicating restored insulin production.

**Table 1:** Clinical trials with published human data. ESC: embryonic stem cell; CiPSC: chemically induced pluripotent stem cell; T1D: type 1 diabetes; Treg: T regulatory cells; HbA1c: glycosylated hemoglobin.

Trial name and sponsor	Year	Clinical trial registration	Phase and status	Study design	Intervention type	Participants (n)	Primary endpoints	Safety outcomes	Efficacy outcomes
ViaCyte PEC-01 Program (ViaCyte Inc.) (Ramzy et al., 2021) [3]	2021	NCT03163511	Phase I/II ongoing	Open-label, first-in-human, multicenter; subcutaneous macroencapsulation device with systemic immunosuppression	ESC-derived pancreatic endoderm cells in a non-immunoprotective macroencapsulation device; requires immunosuppression	15 (single-site cohort)	Safety, feasibility, and evidence of insulin secretion (C-peptide responses)	Well-tolerated. No teratoma formation. No severe graft-related adverse event. Transient immunosuppression-related immune changes (↑ Treg, ↑ PD-1⁺ cells)	Increased fasting and glucose-responsive C-peptide. Development of mixed meal-stimulated C-peptide secretion. Reduced insulin requirements. Improved time-in-range and reduced hypoglycemia
Vertex VX-880 (Vertex Pharmaceuticals) (Lin et al., 2026) [2]	2025 to 2026	NCT04786262	Phase I/II active	Open-label; ESC-derived islet cells with systemic immunosuppression	ESC-derived, fully differentiated pancreatic islet cells (VX-880); requires immunosuppression	Not publicly disclosed	Restoration of β-cell function and insulin independence	Safety data not fully published in peer-reviewed literature. The company reports an acceptable safety profile	Reported cases achieved insulin independence. Restoration of β-cell function demonstrated. Detailed efficacy data pending peer-reviewed publication
Vertex VX-264 (Vertex Pharmaceuticals) (Lin et al., 2026) [2]	2026	TrialTrove ID: 492,327	Early clinical development	ESC-derived β cells with an immunoprotective approach	ESC-derived β cells with encapsulation or immunoprotection strategy (encapsulation-free immunosuppression approach)	Not publicly disclosed	Not publicly disclosed	Program mentioned in reviews. Specific safety data not yet published	Specific efficacy outcomes not yet published. Program in development
Sernova Cell Pouch System (Sernova Corp.) (Kieffer, 2025) [5]	2018 to 2025	NCT02064309, NCT03513939	Phase I/II ongoing	Open-label, implantable vascularized tissue chamber designed to receive donor islets or stem cell-derived islets	Implantable vascularized tissue chamber (Cell Pouch) designed to promote vascularization and cell engraftment	Not publicly disclosed	Device safety, vascularization, and glycemic outcomes	Device implantation trials ongoing. Safety profile under evaluation. Detailed human safety outcomes pending publication	Designed to promote vascularization and cell engraftment. Detailed human efficacy outcomes pending publication
βAir Bioartificial Pancreas (Beta-O2 Technologies) (Carlsson et al., 2018) [42]	2018	NCT02064309	Phase I/II completed	Open-label; macroencapsulation device with oxygen supply	Macroencapsulation device with oxygen supply (βAir); transplanted with human donor islets (potentially adaptable for stem cell-derived islets)	7 patients (T1D)	Safety and feasibility of macroencapsulated islet transplantation with oxygen supply	The device was well tolerated. No device-related serious adverse events. Some patients experienced pericapsular fibrosis	Detectable C-peptide in some patients. Limited insulin independence. Demonstrates proof-of-concept for oxygenated macroencapsulation
Autologous CiPSC-Derived Islet Transplantation (Peking University, China) (Wang et al., 2024) [10]	2024	ChiCTR2300072200	Phase I preliminary results	First-in-human, open-label; autologous CiPSC-derived islets transplanted beneath the abdominal anterior rectus sheath; no encapsulation device	Autologous CiPSC-derived islets	1 (preliminary single-patient report)	Feasibility, safety, and metabolic control of autologous CiPSC-islet engraftment	No transplant-related abnormalities at 1-year follow-up. No safety signals or graft-related pathology. No teratoma formation	Sustained insulin independence beginning on day 75 post-transplant. Time-in-target range: 43.18% (baseline) → 96.21% (month 4) → >98% (sustained). HbA1c decreased to ~5% and remained stable at 1 year. All clinical endpoints were met
Phase 3 Deceased Donor Islet Transplantation (Multiple centers - CIT Consortium) (Hering et al., 2016) [43]	2016	NCT00434811, NCT00468117	Phase III completed	Randomized controlled trial; deceased donor human islets with systemic immunosuppression (Edmonton protocol)	Deceased donor human islets with systemic immunosuppression	48 patients (intensive therapy group)	Restoration of glycemic stability, protection from severe hypoglycemia, and insulin independence	Acceptable safety profile in high-risk populations. Immunosuppression-related adverse events (infections, anemia). No procedure-related deaths	HbA1c reduction: 7.7% → 6.3% at 1 year. Insulin independence: 87.5% at day 75, 71% at 1 year, 52% at 2 years. Elimination of severe hypoglycemic events. Persistent C-peptide production benefits glycemic control

ViaCyte Clinical Trials: PEC-Encap and PEC-Direct

ViaCyte has been a pioneer in translating stem cell-derived β-cell therapies to clinical testing, with multiple trials evaluating ESC-derived pancreatic progenitor cells in different delivery formats. The PEC-Encap device, a macroencapsulation system designed for subcutaneous implantation, was evaluated in an open-label phase 1/2 trial in patients with T1D [9]. This trial demonstrated the safety and feasibility of transplanting macro-encapsulated human stem cell-derived pancreatic endoderm cells, with no reports of teratoma formation or severe graft-related adverse events [9].

After one year, 15 patients showed increased fasting C-peptide levels, increased glucose-responsive C-peptide, and developed mixed meal-stimulated C-peptide secretion [3]. Immunosuppression-related transient increases in circulating regulatory T cells and PD1 were observed [3]. Explanted grafts contained mature β-cells, confirming successful in vivo maturation of the transplanted progenitor cells [5]. However, efficacy was limited by inadequate vascularization and fibrotic encapsulation of the device, which impaired long-term function [28].

To address these limitations, ViaCyte developed the PEC-Direct approach, which involves transplanting the same pancreatic progenitor cells without an immunoprotective device, combined with systemic immunosuppression [44]. This strategy aims to maximize vascularization and cell survival while accepting the requirement for immunosuppression. Early results from PEC-Direct trials have shown more robust C-peptide production compared to PEC-Encap, though comprehensive efficacy data are still emerging [45].

The ViaCyte trials have provided invaluable insights into the challenges of macroencapsulation, the feasibility of in vivo maturation of pancreatic progenitors, and the safety profile of stem cell-derived therapies. These studies have established important precedents for regulatory approval and have informed the design of subsequent clinical trials by other companies [1].

Vertex Pharmaceuticals VX-880 Program

Vertex Pharmaceuticals’ VX-880 program represents one of the most successful stem cell-derived β-cell therapies to date, achieving insulin independence in multiple patients with T1D [2]. VX-880 consists of fully differentiated, functional islet cells derived from a single master cell bank of ESCs, delivered via portal vein infusion with systemic immunosuppression [46].

The VX-880 program has achieved insulin independence in treated patients, representing a major milestone in the field [2]. Clinical trials have consistently demonstrated restoration of insulin independence in immunosuppressed recipients, with patients exhibiting glucose-responsive C-peptide secretion and improved glycemic control [3]. The use of fully differentiated islet cells, rather than progenitors, may contribute to the more rapid functional outcomes observed with VX-880 compared to progenitor-based approaches [47].

Building on the success of VX-880, Vertex is developing VX-264, which incorporates encapsulation technology to eliminate the need for systemic immunosuppression [48]. This next-generation product aims to combine the functional efficacy of VX-880 with immunoprotection, potentially expanding the eligible patient population to include those for whom immunosuppression is contraindicated [49]. Early-stage clinical trials of VX-264 are ongoing, with results eagerly anticipated by the field [50].

The Vertex program demonstrates the potential of stem cell-derived therapies to achieve outcomes comparable to or exceeding those of donor islet transplantation. However, the requirement for portal vein delivery and systemic immunosuppression in VX-880 limits its applicability, underscoring the importance of developing effective encapsulation or immune-evasion strategies [51].

CiPSC-Derived Islet Transplantation: The First Functional Cure

In 2024, a groundbreaking clinical trial reported the first functional cure of T1D using autologous CiPSC-derived islets [10]. This phase I trial (ChiCTR2300072200) involved transplantation of patient-specific islets beneath the abdominal anterior rectus sheath in a 25-year-old patient with T1D [10]. The choice of this implantation site was based on its accessibility, vascularization potential, and ease of monitoring [52].

The outcomes were remarkable: the patient achieved sustained insulin independence 75 days post-transplantation, with time-in-target glycemic range increasing from 43.18% pre-transplant to 96.21% by month four and exceeding 98% at one year [10]. Glycated hemoglobin decreased to non-diabetic levels (approximately 5%), and all study endpoints were met at one year without transplant-related abnormalities [10]. This represents the most successful clinical outcome reported to date for stem cell-derived β-cell therapy.

Several factors may have contributed to this exceptional result. The use of autologous CiPSC-derived cells minimizes alloimmune rejection risk, though immunosuppression was still employed to address potential autoimmune responses [7]. The abdominal anterior rectus sheath implantation site may provide favorable vascularization and mechanical support compared to subcutaneous sites [41]. The use of fully differentiated islet clusters, rather than progenitors, likely enabled more rapid functional engraftment [53].

This landmark study demonstrates the feasibility of achieving durable insulin independence through stem cell-derived β-cell therapy and validates the CiPSC approach for autologous cell replacement. However, important questions remain regarding the scalability of patient-specific cell generation, the long-term durability of graft function, and whether similar outcomes can be achieved with allogeneic cells combined with effective immunoprotection [54].

Other Emerging Clinical Trials

Beyond the major programs described above, numerous other clinical trials are evaluating stem cell-derived β-cell therapies with various combinations of cell sources, differentiation protocols, delivery methods, and immunoprotection strategies [2]. The Sernova Cell Pouch System, for example, employs a subcutaneous implantable device that is prevascularized before cell loading, aiming to address the vascularization challenges observed with other macroencapsulation approaches [42].

Mesenchymal stem cell therapies represent a complementary approach that has been evaluated across phase 1 to 3 trials [2]. Rather than directly replacing β-cells, mesenchymal stem cells provide immunomodulatory and metabolic support that may preserve residual β-cell function and modulate autoimmune responses [55]. Clinical trials have shown that mesenchymal stem cell therapies can improve glycemic control and β-cell preservation, underscoring their potential as an adjunctive or standalone treatment [2].

Several trials are also evaluating xenogeneic (porcine) islet transplantation, which offers an alternative solution to the donor shortage problem [56]. While xenotransplantation faces unique immunological and regulatory challenges, recent advances in the genetic engineering of pigs to reduce immunogenicity have renewed interest in this approach [57]. The convergence of multiple technological platforms, i.e., stem cell-derived islets, encapsulation, hypoimmune engineering, and xenotransplantation, reflects the diversity of strategies being pursued to achieve the common goal of safe, effective, and scalable β-cell replacement for T1D [58].

Comparative analysis: Key findings across studies

Efficacy Outcomes: C-peptide Secretion and Insulin Independence

A critical metric for evaluating β-cell replacement therapies is the restoration of endogenous insulin production, typically measured by C-peptide levels. C-peptide, a byproduct of insulin processing, provides a direct measure of β-cell function that is not confounded by exogenous insulin administration [59]. Across recent clinical trials, the ability to restore detectable and glucose-responsive C-peptide secretion has been consistently demonstrated, though the magnitude and kinetics vary considerably [3,9,10].

The ViaCyte PEC-Encap trials showed increased fasting and glucose-responsive C-peptide levels, with development of mixed meal-stimulated C-peptide secretion over 12 months [3]. However, C-peptide levels remained relatively modest, and no patients achieved insulin independence with the encapsulated approach [9]. In contrast, the Vertex VX-880 program achieved insulin independence in multiple patients, with robust C-peptide production comparable to successful donor islet transplantation [2]. The CiPSC-derived islet trial achieved the most dramatic outcome, with sustained insulin independence and near-normal glycemic control maintained for at least one year [10].

These differences in efficacy likely reflect multiple factors, including the maturity of transplanted cells (progenitors vs. fully differentiated islets), the cell dose delivered, the implantation site and its vascularization, and the presence or absence of physical barriers to nutrient exchange [60]. The superior outcomes with non-encapsulated approaches (VX-880 and CiPSC islets) suggest that current encapsulation technologies still impose significant limitations on cell function, though they offer the critical advantage of eliminating or reducing immunosuppression requirements [61].

Time-in-target glycemic range represents another important efficacy metric that captures the practical impact of therapy on daily glycemic control. The CiPSC trial demonstrated improvement from 43.18% to over 98% time-in-target range, representing near-normalization of glycemic control [10]. Such outcomes, if reproducible across larger patient populations, would represent a transformative advance in T1D management, potentially eliminating the burden of insulin therapy and reducing long-term complications [62].

Safety Profile and Adverse Events

Safety has been a paramount concern in stem cell-derived β-cell trials, given the theoretical risks of teratoma formation from residual undifferentiated cells, immune-mediated complications, and device-related adverse events [63]. Reassuringly, clinical trials to date have demonstrated acceptable safety profiles, with no reports of teratoma formation in any published study [3,9,10]. This likely reflects improvements in differentiation protocols that achieve high purity of pancreatic lineages and incorporate quality control measures to detect and eliminate undifferentiated cells [64].

Device-related complications have been observed in macroencapsulation trials, primarily consisting of fibrotic overgrowth and inadequate vascularization [28]. These issues, while not life-threatening, have limited efficacy and, in some cases, necessitated device removal [65]. Strategies to mitigate fibrosis, including device design modifications, anti-fibrotic coatings, and prevascularization approaches, are under active investigation [29].

Immunosuppression-related adverse events have been reported in trials employing systemic immunosuppression, consistent with the known risks of these medications [3]. These include increased susceptibility to infections, metabolic effects, and potential nephrotoxicity [66]. The observation of transient increases in regulatory T cells and PD1 expression in some trials suggests that immunosuppressive regimens may have immunomodulatory effects beyond simple immune suppression [3]. However, the long-term safety of chronic immunosuppression remains a significant concern, particularly for younger patients with decades of life expectancy [67].

The CiPSC trial reported no transplant-related abnormalities at one year, representing an excellent safety outcome [10]. However, longer follow-up will be essential to assess the durability of graft function and to detect any late-onset complications [68]. The field has generally adopted a cautious approach to dose escalation and patient selection, prioritizing safety while gathering efficacy data [69].

Immunosuppression Requirements

A central goal of encapsulation and hypoimmune engineering strategies is to eliminate or minimize the need for systemic immunosuppression. Current clinical trials span a spectrum from full systemic immunosuppression (VX-880, CiPSC trial) to no immunosuppression (some encapsulation trials) [2,10]. The trade-offs between these approaches are becoming increasingly clear: non-immunosuppressed encapsulated approaches offer superior safety profiles but have thus far achieved limited efficacy, while immunosuppressed non-encapsulated approaches achieve robust efficacy but carry the risks of chronic immunosuppression [70].

The ViaCyte PEC-Encap device was designed to eliminate the need for immunosuppression, and early trials were conducted without systemic immunosuppression [9]. However, limited efficacy led to subsequent trials incorporating immunosuppression to enhance outcomes [71]. This evolution reflects the ongoing challenge of achieving adequate immunoprotection through encapsulation alone, particularly in the face of both alloimmune and autoimmune responses [72].

The use of immunosuppression in the CiPSC trial, despite the autologous nature of the cells, highlights the persistent challenge of autoimmune recurrence in T1D [7]. Even patient-specific cells remain vulnerable to the autoimmune process that destroyed native β-cells, necessitating strategies to protect against autoimmune attack [73]. This underscores the importance of developing effective local immunomodulation strategies that can address both alloimmune and autoimmune rejection without systemic immunosuppression [74].

Future trials evaluating hypoimmune engineered cells and advanced encapsulation technologies will be critical for determining whether immunosuppression-free β-cell replacement is achievable [15]. The ideal therapy would combine the efficacy of current immunosuppressed approaches with the safety profile of immunosuppression-free strategies, potentially through synergistic combinations of multiple immunoprotection modalities [75].

Discussion: Challenges and future directions

Cell-based therapies for T1D have emerged as a promising avenue to address the limitations of current treatments, such as insulin injections and islet transplantation. Despite significant advancements, several challenges remain, including immune rejection, scalability, and the need for effective encapsulation strategies. This section explores the current challenges and future directions in the development of cell-based therapies for T1D.

Overcoming Fibrosis and Vascularization Barriers

Fibrotic encapsulation and inadequate vascularization have emerged as critical barriers to the success of encapsulated β-cell therapies [28]. The foreign body response to implanted devices triggers a cascade of inflammatory and fibrotic processes that can isolate the device from the host vasculature, impairing nutrient and oxygen delivery and waste removal. This problem is particularly acute for macroencapsulation devices, which present a large surface area and may be more prone to fibrosis than smaller microencapsulated constructs.

Multiple strategies are being pursued to mitigate fibrosis and enhance vascularization. Device design modifications, including incorporation of vascular portals, surface texturing, and optimized membrane architecture, aim to promote controlled vascularization while maintaining immunoprotection [5,29]. Biomaterial modifications, such as coating with anti-fibrotic molecules or incorporating pro-angiogenic factors, represent another approach [6,12]. Prevascularization strategies, in which devices are implanted and allowed to vascularize before cell loading, have shown promise in preclinical studies and are being evaluated clinically [22].

The choice of implantation site significantly influences vascularization and fibrosis outcomes. Subcutaneous sites, while convenient and accessible, may be suboptimal due to limited vascularity and propensity for fibrosis. Alternative sites under investigation include the omental bursa, which offers rich vascularization and immunomodulatory properties, and the abdominal anterior rectus sheath, which was successfully employed in the CiPSC trial [10,16]. Intraperitoneal delivery and portal vein infusion offer excellent vascularization but present technical challenges and safety concerns.

Emerging technologies such as 3D bioprinting may enable the creation of more sophisticated encapsulation constructs with optimized architecture for vascularization and nutrient exchange [5]. The integration of multiple strategies, including optimized device design, bioactive materials, prevascularization, and strategic site selection, will likely be necessary to fully overcome the vascularization and fibrosis challenges that have limited encapsulation success to date.

Scalability and Manufacturing Considerations

The translation of stem cell-derived β-cell therapies from small-scale clinical trials to widespread clinical use will require addressing substantial manufacturing and scalability challenges. Current differentiation protocols typically employ multi-week processes with multiple media changes, growth factor additions, and quality control steps, making them labor-intensive and expensive. The generation of sufficient cell numbers for clinical transplantation (typically hundreds of millions to billions of cells per patient) requires large-scale culture systems and stringent quality control.

Several approaches are being pursued to improve manufacturing scalability. Suspension culture systems and bioreactors can enable larger-scale cell production compared to traditional adherent culture methods. The development of defined, xeno-free culture media and recombinant growth factors reduces variability and regulatory complexity. Automation of culture processes and quality control testing can improve reproducibility and reduce costs.

The choice between autologous and allogeneic cell sources has profound implications for manufacturing scalability. Autologous approaches, while offering immunological advantages, require generating a separate cell product for each patient, with associated time delays and costs [10]. Allogeneic approaches, using cells from a single master cell bank, enable economies of scale and off-the-shelf availability but require effective immunoprotection strategies. The field is increasingly converging on allogeneic approaches as more practical for widespread clinical deployment, though autologous therapies may retain a role for select patients.

Cryopreservation and storage of differentiated cells or progenitors is another critical consideration for clinical logistics. The ability to generate large batches of cells, cryopreserve them, and ship them to clinical sites enables centralized manufacturing and quality control. However, cryopreservation can affect cell viability and function, necessitating optimization of freezing and thawing protocols.

Regulatory considerations for cell therapy manufacturing are complex and evolving. Good manufacturing practice requirements for cell production, quality control testing for safety and potency, and lot release criteria must all be established and validated. The regulatory pathway for stem cell-derived therapies is still being defined, with ongoing dialogue between developers and regulatory agencies to establish appropriate standards.

Regulatory Pathways and Clinical Translation

The regulatory landscape for stem cell-derived β-cell therapies is complex, involving considerations of both the cell product and, in many cases, the delivery device. In the United States, these therapies are regulated as biological products by the Food and Drug Administration’s Center for Biologics Evaluation and Research, with additional oversight from the Center for Devices and Radiological Health for device components [76]. The path to regulatory approval typically involves sequential phase 1, 2, and 3 clinical trials demonstrating safety and efficacy in progressively larger patient populations.

A critical regulatory milestone is the Biologics License Application, which is required for commercial marketing of cell therapy products [17]. The Biologics License Application process requires comprehensive data on manufacturing, quality control, preclinical studies, and clinical trial results. Recent reviews have emphasized the importance of early engagement with regulatory agencies to align on development plans, endpoints, and approval criteria [17].

The regulatory pathway for combination products (cells plus devices) presents additional complexity, requiring coordination between different regulatory divisions and consideration of both biological and device-specific requirements. The designation of a lead center (typically the Center for Biologics Evaluation and Research for cell-device combinations) helps streamline the review process.

Harmonization of regulatory standards across different countries and regions is an ongoing challenge that affects the global development and deployment of cell therapies [2]. Different regulatory agencies may have varying requirements for preclinical data, clinical trial design, and manufacturing standards. International collaboration and alignment of regulatory frameworks could accelerate the development and availability of these therapies worldwide.

Patient selection criteria for clinical trials and eventual clinical use represent another important consideration. Current trials have generally focused on patients with established T1D, hypoglycemic unawareness, or brittle diabetes, populations with the greatest unmet need and most favorable risk-benefit profiles. As safety and efficacy data accumulate, expansion to broader patient populations may be considered.

Toward Immunosuppression-Free Therapies

The ultimate goal of the field is to achieve effective β-cell replacement without the need for chronic systemic immunosuppression. This goal is motivated by the significant morbidity associated with immunosuppression and the desire to make cell therapy accessible to the broader T1D population, including children and young adults for whom the risks of immunosuppression may outweigh the benefits.

Multiple complementary strategies are being pursued to achieve immunosuppression-free therapy. Physical encapsulation technologies continue to evolve, with improvements in materials, device design, and implantation strategies aimed at achieving robust immunoprotection while maintaining cell function [12,23]. The incorporation of bioactive molecules that actively modulate local immune responses represents a promising enhancement to passive barrier approaches [11].

Hypoimmune cell engineering, through deletion or modification of HLA genes and expression of immunomodulatory molecules, offers an alternative or complementary approach [15,24]. Early clinical trials of gene-edited immune-evasive cells are now underway, which will provide critical data on the safety and efficacy of this strategy [2]. The combination of hypoimmune cells with encapsulation may provide synergistic immunoprotection that exceeds either approach alone.

Tolerance induction strategies, aimed at specifically suppressing immune responses to transplanted cells without global immunosuppression, represent another frontier. Approaches under investigation include co-transplantation of regulatory T cells, use of tolerogenic dendritic cells, and administration of immunomodulatory biologics that promote tolerance. The integration of cell therapy with emerging immunotherapies for T1D, such as anti-CD3 antibodies or antigen-specific tolerance induction, could potentially address both β-cell replacement and autoimmune disease modification [15].

The path to immunosuppression-free therapy will likely require iterative refinement of multiple technologies and their integration into optimized combination approaches. Success will depend on continued innovation in stem cell biology, biomaterials science, immunology, and bioengineering, along with carefully designed clinical trials to evaluate these complex interventions.

## Conclusions

Cell-based therapies for T1D have progressed from theoretical concept to clinical reality, with multiple trials now demonstrating proof of concept for restoration of insulin independence through stem cell-derived β-cell replacement. The field has achieved remarkable milestones, including the first functional cure of T1D using autologous CiPSC-derived islets and consistent demonstration of insulin independence with allogeneic ESC-derived cells in immunosuppressed recipients. These achievements validate decades of research in stem cell biology, bioengineering, and transplantation immunology.

However, significant challenges remain before cell-based therapies can become routine clinical practice. The requirement for systemic immunosuppression in the most successful trials to date limits applicability to a subset of patients with severe disease. Encapsulation technologies, while promising in principle, have not yet achieved the combination of robust immunoprotection and adequate cell function necessary for widespread clinical use. Fibrosis, vascularization, long-term graft survival, and manufacturing scalability all require further innovation and optimization. For the millions of individuals living with T1D worldwide, these advances offer renewed hope for a future free from the burden of insulin therapy and the complications of imperfect glycemic control. The transformation of T1D from a chronic disease requiring lifelong management to a curable condition through cell-based therapy represents one of the most exciting frontiers in regenerative medicine and offers a compelling vision for the future of diabetes care.
